# Ischemic Nephropaty: The Role of the Renal Artery Stenosis Revascularization on Renal Stem Cells

**DOI:** 10.3390/medicina57090944

**Published:** 2021-09-08

**Authors:** Rosario Cianci, Adolfo Marco Perrotta, Antonietta Gigante, Federica Errigo, Claudio Ferri, Eleonora Cianci, Mariadelina Simeoni, Sandro Mazzaferro, Silvia Lai

**Affiliations:** 1 Nephrologic Unit, Department of Translational and Precision Medicine, University of Rome ‘La Sapienza’, 00185 Rome, Italy; rosario.cianci@uniroma1.it (R.C.); antonietta.gigante@uniroma1.it (A.G.); federica.errigo@uniroma1.it (F.E.); eleonoracianci@hotmail.it (E.C.); sandro.mazzaferro@uniroma1.it (S.M.); silvia.lai@uniroma1.it (S.L.); 2 Internal Medicine and Nephrology Unit—San Salvatore Hospital, Department of Clinical Medicine and Public Health, University of L’Aquila, 67100 L’Aquila, Italy; claudio.ferri@univaq.it; 3 Department of Translational Medical Sciences, University of Campania ‘Luigi Vanvitelli’, 80138 Napoli, Italy; mariadelina.simeoni@unicampania.it

**Keywords:** percutaneous transluminal renal angioplasty, renal artery stenosis, renovascular hypertension, staminal renal cells

## Abstract

We report the case of a 65-year-old man with acute GFR decline to 37 mL/min and uncontrolled high blood pressure. He was suspected for renovascular hypertension and underwent a renal color Doppler ultrasound scan that detected a bilateral atherosclerotic renal artery stenosis. A digital selective angiography by percutaneous transluminal angioplasty and stenting (PTRAs) was successfully performed. Blood pressure rapidly normalized, GFR increased within a few days, and proteinuria disappeared thereafter. These clinical goals were accompanied by a significant increase of circulating renal stem cells (RSC) and a slight increase of resistive index (RI) in both kidneys. This single observation suggests the need for extensive studies aimed at evaluating the predictive power of RI and RSC in detecting post-ischemic renal repair mechanisms.

## 1. Introduction

Long term ischemic nephropathy (IN) is associated with an increased risk of progressive renal function decline. Due to the aging and the high prevalence of diabetes mellitus type 2, dyslipidemia, and hypertension in the general population, the most common cause of severe IN is related to atherosclerotic renal artery stenosis (RAS) rather than fibromuscular dysplasia [1,2]. Percutaneous transluminal renal angioplasty (PTRA) with or without stenting is the gold standard treatment of RAS [3]. However, therapeutic success depends on the correct timing of revascularization that should be performed when kidney damage is still reversible [1]. On the other hand, we lack thus far highly sensitive and specific diagnostic tools able to precisely predict the renal outcome after PTRA. Randomized controlled trials comparing medical therapy alone with the combination of medical therapy and PTRA failed to show a benefit of PTRA [4]. However, none of the studies conducted on humans have focused on the post-revascularization factors able to repair organ damage. Renal resistive index (RRI) could reflect the full activation of the compensatory renal blood flow (RBF) autoregulation as the expression of a preserved microvascular representation [5].

Coming to possible repairing factors, several reports on the role of mesenchymal stem cells in renal tissue regeneration after kidney reperfusion have been conducted on animal models and showed promising results. In the adult human kidney, CD133+, CD24− and CD45− cells are progenitors arranged in a precise sequence within Bowman’s capsule and exhibit heterogeneous potential for differentiation and regeneration. Here, we present a case report that opens the perspective for circulating CD133+, CD24+, and CD45− dosage for being a valid prognostic tool able to predict renal outcome after PTRA [1,6,7]. 

## 2. Case Presentation

A 65-year-old hypertensive man with glomerular filtrate rate (GFR) reduction to 37 mL/min/1.73 m^2^ and proteinuria in the nephrotic range was referred to our Hypertension Center with suspicion of renovascular hypertension.

His past medical history included a one-year history of hypertension resistant to calcium channel blockers (amlodipine 10 mg, daily) and Beta-blocker with poor blood pressure (BP) control (mean day time BP value of 170/100 mmHg at the 24 h BP monitoring). After the introduction of an ARB (Irbesartan 300 mg daily), he had a little improvement in blood pressure while GFR quickly declined from 37 to 26 mL/min.

A color Doppler ultrasound (CDU) scan was promptly performed and detected a bilateral RAS in the ostial portion of both renal arteries. Significantly high systolic peak velocity of (380 cm/s right, 350 cm/s left) were detected, indicating bilateral hemodynamic stenosis. The renal resistive index was bilaterally normal (0.64).

Observing severe clinical symptoms and renal function worsening, an angiography was promptly performed with bilateral stenting by the use of 6 × 19 mm Metal Renal Dynamic Stents.

In the overall procedure, no complications were observed, and a BP decrease to 145/80 mmHg was registered within a few hours, even GFR increased after 48 h. At the 3 and 12-month follow-up, on suspension of anti-hypertensive agents, BP remained well controlled, and proteinuria gradually decreased (Table 1).

Based on several reports attributing to specific bone marrow mesenchymal cells, the ability to repair ischemic kidney tissue after revascularization in animals [6,7], we recently added the count of circulating RSC before (T0) and after revascularization (T1) to our internal protocol. Blood samples were obtained by antecubital venipuncture, and a volume of 100 μL of whole blood was stained with combinations of mAb against human CD24, CD133, and CD45. MAb was conjugated with fluorescein isothiocyanate (FITC), phycoerythrin (PE), or allophycocyanin (APC) (all from eBiosciences). Unstained cells were used as a negative control. After 30 min incubation at 4 °C of antibodies, cells were subjected to red blood cells lysis and analyzed by a FACS Calibur (Becton and Dickinson). A total of 100,000 events/samples were run, and data were analyzed using the Cell Quest Pro software (BD BioSciences). RSC population was defined as CD45−CD24+CD133+.

Cytometry analysis showed that the percentage of RSC was 1.35% (1354 CD45−CD24+CD133+ cells/100.000 events) at T0. The percentage of RSC rose to 4.3% (4321 CD45−CD24+CD133+ cells/100.000 events) after revascularization. Notably, this significant RSC increase was followed by the described positive renal and hemodynamic outcomes.

## 3. Discussion

Atherosclerotic RAS is the most common cause of ischemic nephropathy in western countries [1]. Hypoxia is the noxa that leads to renal damage and then to chronic kidney disease. Low blood oxygen levels carry out to a parenchymal fibrotic as in ischemia–reperfusion injury model. Reduced renal microvascular density due to fibrosis causes tubulointerstitial damage leading to CKD [8]. Eirin A. et al. reported that gradual reduction in renal blood flow (RBF) results in kidney volume ad function loss, reversible only with a prompt renin–angiotensin system activation. The same authors fixed to 30–40% RBF reduction, the cut-off for reversibility of ischemic renal damage. Over that, the kidney adaptive response is less effective. This is what happens in severe RAS. This condition is accompanied by high levels of circulating pro-inflammatory cytokines. Renal damage progression reflects microvascular rarefication, and it is not reversible even after large-vessel blood flow restoration [9]. This evidence would suggest adjunctive therapeutic measures, beyond renal revascularization, as mitochondrial protection, angiogenic cytokine therapy, stem cell-based regeneration.

Several studies have demonstrated a significant expression of mesenchymal stem cells in almost all organs and tissues [6,7,10,11]. Bone marrow mesenchymal stem cells are able to differentiate into multiple tissues, such as osteoblasts, chondrocytes, neural cells, blood cells, and endothelium [6]. Gupta et al. reported the expression of the embryonic stem cells markers Oct4 and Pax-2 in rat kidneys, demonstrating the presence of a resident multipotent stem cells population in renal tissue. Intravenous or trans-capsular inoculation of these cells, after ischemia-reperfusion injury, induced their tubular differentiation [9].

Surprisingly, no studies in humans have been published thus far for ascertaining the factors triggering renal repair in post-ischemic nephropathy.

In our patient with bilateral RAS, we observed a rapid post-revascularization improvement of renal outcomes (Table 1), normalization of blood pressure, proteinuria reduction, and eGFR increase. All these factors are accompanied by an increase in circulating RSC.

We have thought that the rapid increase at T1 of the number of stem cells and the following decrease, after 90 days, to basal levels is suggestive. We hypothesized that this evidence could suggest the differentiation of the RSC into tubular cells and/or podocytes. The improvement of clinical parameters could support the hypothesis. However, it could also be related to the hemodynamic changes induced by the reperfusion.

Another interesting observation offered by our case report is represented by the slight increase in RI after revascularization, which could be explained as an expression of the restoration of intrarenal hemodynamics after interruption of compensatory vasodilation due to the ischemia.

In conclusion, we believe that our single observation sets the stage for broader experimental and clinical studies to assess our hypothesis. These could lead to detecting new indicators for the selection of patients expected to have better post-reperfusion outcomes.

## Figures and Tables

**Table 1:** Changes of lab parameters before (T0), 10 days after PTRAs (T1), and 3 months after PTRAs (T2).

Parameter	Pre-Revascularization (T0)	10 Days after Revascularization (T1)	3 Months after Revascularization (T2)
Creatinine (mg/dL)	1.85	1.5	1.47
CKD EPI GFR (mL/min/1.73 m^2^)	37		48
BUN (mg/dL)	34	25	28
Na^+^ (mmol/L)	140	138	140
K^+^ (mmol/L)	5	3.7	4.6
Ca^2+^ (mg/dL)	9.6	9.1	9.2
PO_4_^−^ (mg/dL)	3.8	3.1	3
Cl− (mmol/L)	101	99	100
Mg^2+^ (mg/dL)	2.2	1.9	2
Uric acid	6.7	4.9	4.8
Hemoglobin (g/dL)	17.5	15.7	15.5
RBC (*10^3^/mmc)	5570	5450	5100
Platelets (*10^3^/mmc)	186	163	147
24 h Proteinuria (g/24 h)	1.6	0.21	0.25
Stem Cell CD24+CD45−CD133+ (%)	2.34	6	3

## Data Availability

No new data were created or analyzed in this study. Data sharing is not applicable to this article.
